# Optimizing planting density and nitrogen application to enhance maize yield while mitigating carbon emission in southern Xinjiang

**DOI:** 10.3389/fpls.2026.1809981

**Published:** 2026-04-27

**Authors:** Wandong Han, Zhengjun Cui, Wenfeng Li, Ling Li, Bingqin Qi, Dan Zhang, Xinning Luo, Jinbin Wang

**Affiliations:** 1 College of Agriculture, Tarim University, Alar, China; 2 Key Laboratory of Genetic Improvement and Efficient Production for Specialty Crops in Arid Southern Xinjiang of Xinjiang Corps, Tarim University, Alar, China; 3 College of Information Engineering, Tarim University, Alar, China

**Keywords:** carbon balance, maize yield, nitrogen rate, planting density, soil carbon emission

## Abstract

**Introduction:**

Optimizing planting density and nitrogen application rate are key factors for increasing maize yield, but they also significantly affect farmland CO_2_ emissions. The appropriate nitrogen application rate and planting density for achieving high-yield and low-carbon maize production in southern Xinjiang remain to be determined.

**Methods:**

A field experiment was conducted to investigate the effects of planting density and nitrogen rate on soil CO_2_ emissions, carbon balance, and maize yield in 2024 and 2025. The treatments included four nitrogen rates (0 (N0), 180 (N1), 270 (N2), 360 (N3) kg N ha^-1^) and four planting densities (90,000 (D1), 120,000 (D2), 150,000 (D3), 180,000 (D4) plants ha^-1^).

**Results:**

The results showed that soil respiration rate exhibits a distinct unimodal curve, reaching its maximum during the maize flowering stage, and showed a highly significant positive correlation with soil temperature. Soil CO_2_ emission under N0, N1, and N2 treatments were lower than under the N3 treatment, under the D1, D2, and D3 treatments were lower than under the D4 treatment. Compared to N0 and N1, the N2 increased grain yield by 47.9% and 15.8%, increased carbon emission efficiency by 32.6% and 8.9%, no significant between N2 and N3. Compared to the D1, the D2 and D3 increased grain yield by 7.5% and 11.9%, carbon emission efficiency under the D1, D2, and D3 increased by 9.1%, 10.2% and 8.4%, compared to the D4, respectively. The N2 treatment increased NEP compared to N0 and N1, but no significant with N3. NEP under D2 was higher than under D4 in 2024, no significant difference was found among D2, D3, and D4 in 2025.

**Conclusion:**

In conclusion, applying nitrogen at approximately 270 kg ha^-1^ and adopting planting densities of 120,000–150,000 plants ha^-1^ under maize fields in southern Xinjiang can ensure high yields while mitigating CO₂ emissions. This approach also optimizes carbon emission efficiency and enhances the carbon sequestration potential of farmland, representing an optimized strategy for achieving green and intensive agricultural production.

## 1 Introduction

Agricultural ecosystems play a dual role in the global carbon cycle, acting as both a source and a sink of atmospheric carbon dioxide (CO_2_) (Roth et al., 2023). Soil respiration, a primary pathway for CO_2_ release from terrestrial ecosystems, is significantly influenced by field management practices (Ran et al., 2017). In intensive agricultural production, optimizing these practices is crucial for balancing crop productivity enhancement with greenhouse gas mitigation. South Xinjiang is a typical arid region for agricultural production, the abundant sunlight and heat resources in this region provide favorable conditions for maize production (Bian et al., 2024). Most of the maize in Southern Xinjiang is used as animal feed, playing a significant role in ensuring food security and feed supply (Liang et al., 2025). Maize (Zea mays L.), as a major global food crop, often relies on nitrogen fertilizer inputs and varying planting densities to achieve high yields (Zhang et al., 2021). However, the interactive effects of these two key management factors on soil CO_2_ emissions particularly in arid and semi-arid reclaimed regions have not yet been sufficiently quantified.

The interactive effects of nitrogen fertilization and planting density on soil carbon emission have emerged as a focal point in recent research (Guo et al., 2025). Nitrogen application impact soil respiration rates by directly influences soil biogeochemical processes (Zhou et al., 2014). Planting density dictates root spatial distribution, regulates the soil microenvironment, root respiration, and litter input, and consequently impacts carbon emissions (Chen et al., 2019). Although previous studies have separately examined the independent effects of nitrogen application rate (Yan et al., 2020) or planting density (Yang et al., 2021) on soil CO_2_ flux, their potential synergistic or antagonistic interactions remain complex and context dependent.

Oasis agricultural reclamation zones in arid regions are ecologically fragile and rely heavily on irrigation, representing a critical yet understudied area in related research (Li et al., 2021). Soil carbon dynamics in these regions are highly sensitive to both climatic conditions and management interventions (Tamang et al., 2024). Previous studies in similar environments have often been limited to single−factor experiments (Wang et al., 2019) or narrow management gradients (Yang et al., 2022), constraining the predictive understanding of soil carbon dynamics under varied agronomic practices. Therefore, there is a pressing need to systematically investigate how nitrogen and planting density jointly regulate soil CO_2_ emission dynamics in such water−limited cropping systems.

To address this gap, we conducted a field experiment with four planting densities and four nitrogen levels, to (1) investigate the effects of planting density and nitrogen application rate on soil CO_2_ emissions, (2) quantify the effects of nitrogen rate and planting density on maize yield and carbon balance, and (3) explore the synergistic optimization of high yield and low carbon in maize fields in southern Xinjiang through planting density and nitrogen fertilizer gradient. This research is expected to provide an empirical basis for formulating integrated nitrogen and density management strategies that maintain maize yield while reducing the carbon footprint of intensive production.

## 2 Materials and methods

### 2.1 Site description

This study was conducted during at the South Xinjiang Integration of Industry and Education Practical Training Base of Tarim University, located in Alaer City, Xinjiang Uygur Autonomous Region (Longitude: 81.072005°, Latitude: 40.647242°) in 2024 and 2025. The experimental site has an average elevation of 1000 m, an annual frost-free period of 220 days, and falls within a warm−temperate extreme continental arid desert climate. The mean annual sunshine duration ranges from 2556.3 to 2991.8 hours, with an annual solar radiation of 133.7-146.3 kJ cm^-2^. The mean annual air temperature is 10.5 °C, and the long−term average precipitation ranges between 40.1 and 82.5 mm, while annual evaporation reaches 1876.6-2558.9 mm. Both inter−annual and intra−annual variability in precipitation is high in this region. The available light and water resources at the site can support double−cropping systems per year, which is representative of oasis irrigated agriculture in the arid farming areas of southern Xinjiang. The soil properties in 0–20 cm layer of the experimental field as follows: pH, 8.01; soil organic carbon, 3.5 g kg^-1^, total nitrogen, 0.70 g kg^-1^, available phosphorus, 12.85 mg kg^–1^; and available potassium, 67.95mg kg^-1^.

### 2.2 Experimental design

Based on the drip irrigation under mulch system, a two−factor randomized complete block design was adopted. The main plot factor was nitrogen (N) application rate with four levels: 0 kg N ha^-1^ (N0), 180 kg N ha^-1^ (N1), 270 kg N ha^-1^ (N2), and 360 kg N ha^-1^ (N3). The sub−plot factor was planting density also with four levels: 90,000 plants ha^-1^ (D1), 120,000 plants ha^-1^ (D2), 150,000 plants ha^-1^ (D3), and 180,000 plants ha^-1^ (D4). The combination of four N rates and four planting densities resulted in 16 treatments. Each treatment was replicated three times, totaling 48 plots arranged randomly within three blocks. Each plot measured 44 m^2^ (4.4 m × 10 m). Nitrogen fertilizer was applied as urea (46% N). For all N application treatments, the total N was split into three equal doses: one−third as basal fertilizer at sowing, one−third at the jointing stage, and one−third at the flowering−silking stage. The maize cultivar ‘Xinyu 335’ was used as the test material. Planting followed a four−row per mulch bed pattern, with row spacing configured as 70 cm between wide rows and 40 cm between narrow rows. Sowing was performed using a dibbler in late April, and harvest took place in early September. Manual weeding was carried out during the maize growing season, and all other field management practices followed conventional local practices.

### 2.3 Measurements

#### 2.3.1 Soil moisture and temperature

Soil samples from the 0–10 cm depth were collected to determine gravimetric soil water content using the oven-drying method. Soil temperature was monitored at depths of 5 cm and 10 cm with soil thermometers, with data recorded at 10-day intervals.

#### 2.3.2 Soil respiration rate

Soil total respiration rate (Rs) was measured using an LI-870 open−path CO_2_/H_2_O gas analyzer (LI−COR Inc., Lincoln, NE, USA). The first measurement was conducted after maize sowing, with subsequent measurements taken approximately every 10 days. The exact timing was adjusted slightly based on weather and rainfall conditions. The final measurement was performed after maize harvest. Measurements were taken by placing the soil respiration chamber on a PVC collar (inner diameter: 22 cm; height: 11.5 cm) installed in each plot. The collar was inserted into the soil, leaving 2–3 cm above the ground. One collar was installed per plot, and each chamber measurement was performed once per collar. To minimize soil disturbance, any live plants and litter inside the collar were carefully removed 12 hours prior to each measurement. The plastic film covering the collar area was also removed at that time. After the respiration measurement was completed, the film was replaced. This procedure was repeated for each measurement cycle.

#### 2.3.3 Total CO_2_ emissions

The total soil CO_2_ emissions (CE, kg ha^-1^) were calculated based on the measured soil respiration rates and the measurement frequency, using the following Equation 1 (Fang et al., 2022):

(1) $$CE\;=\;\sum \left[\frac{Ri+1\;+\;Ri}{2}\;\times \;\left(ti+1\;-\;ti\;\right)\;\times \;0.1584\;\times \;24\right]\;\times \;0.2727\;\times \;10$$

where R is the soil respiration rate (μ mol CO_2_ m^-2^ s^-1^), “i+1” and “ i “ represent two consecutive measurement times, and “t” is the number of days after sowing. The constant 0.1584 converts μ mol CO_2_ m^-2^ s^-1^ to g CO_2_ m^-2^ h^-1^, 0.2727 converts g CO_2_ m^-2^ h^-1^ to g C m^-2^ h^-1^, and the factors 24 and 10 are applied to convert the final unit from g C m^-2^ h^-1^ to kg C ha^-1^.

#### 2.3.4 Yield

Maize grain yield was determined based on the actual harvested yield per plot. At harvest, yield was measured separately for each plot. Ten representative plants were selected from each treatment to record key agronomic traits, including the number of ears per plant, the number of kernel rows per ear, and the number of kernels per row. Samples were retained for further analysis after yield measurement.

#### 2.3.5 Carbon emission efficiency

Carbon emission efficiency (CEE, kg kg^-1^) defined as the maize grain yield produced per kilogram of CO_2_ emitted, was calculated using the following Equation 2 (Wang et al., 2023):

(2) $$CEE=\frac{Y}{CE}$$

where *Y* is the maize grain yield (kg ha^-1^) and *CE* represents the cumulative CO_2_ emissions during the maize growing season (kg ha^-1^).

#### 2.3.6 Carbon budget

The carbon budget of the farmland system was assessed using net ecosystem productivity (Equations 3–6) (NEP, kg C ha^-1^), calculated as follows (Kuzyakov, 2002; Yang et al., 2013; Wu et al., 2017; Zhang et al., 2018):

(3) NEP =NPP−Rm

(4) NPP= 0.45×DW

(5) Rm=CE×0.865

(6) $$\text{Cs}=\frac{NEP}{CE}$$

where NPP is the net primary productivity carbon sequestration (kg C ha^-1^), estimated from aboveground biomass; R_m_ represents the carbon emission from soil microbial heterotrophic respiration (kg C ha^-1^); CE denotes the cumulative soil CO_2_ emissions during the maize growing season (kg C ha^-1^); DW is the aboveground biomass of maize (kg ha^-1^); 0.45 is the carbon content coefficient for crop aboveground biomass; 0.865 is the conversion coefficient for soil microbial heterotrophic respiration; and Cs indicates the soil carbon sequestration potential of the ecosystem.

### 2.4 Data analysis

Data were statistically analyzed using Microsoft Excel 2019 and SPSS 27.0. Variance analysis was performed with a general linear model, and multiple comparisons were conducted using Duncan’s multiple range test (α = 0.05). Figures were prepared with Origin 2024 software.

## 3 Results

### 3.1 Soil temperature

**Figure 1:**
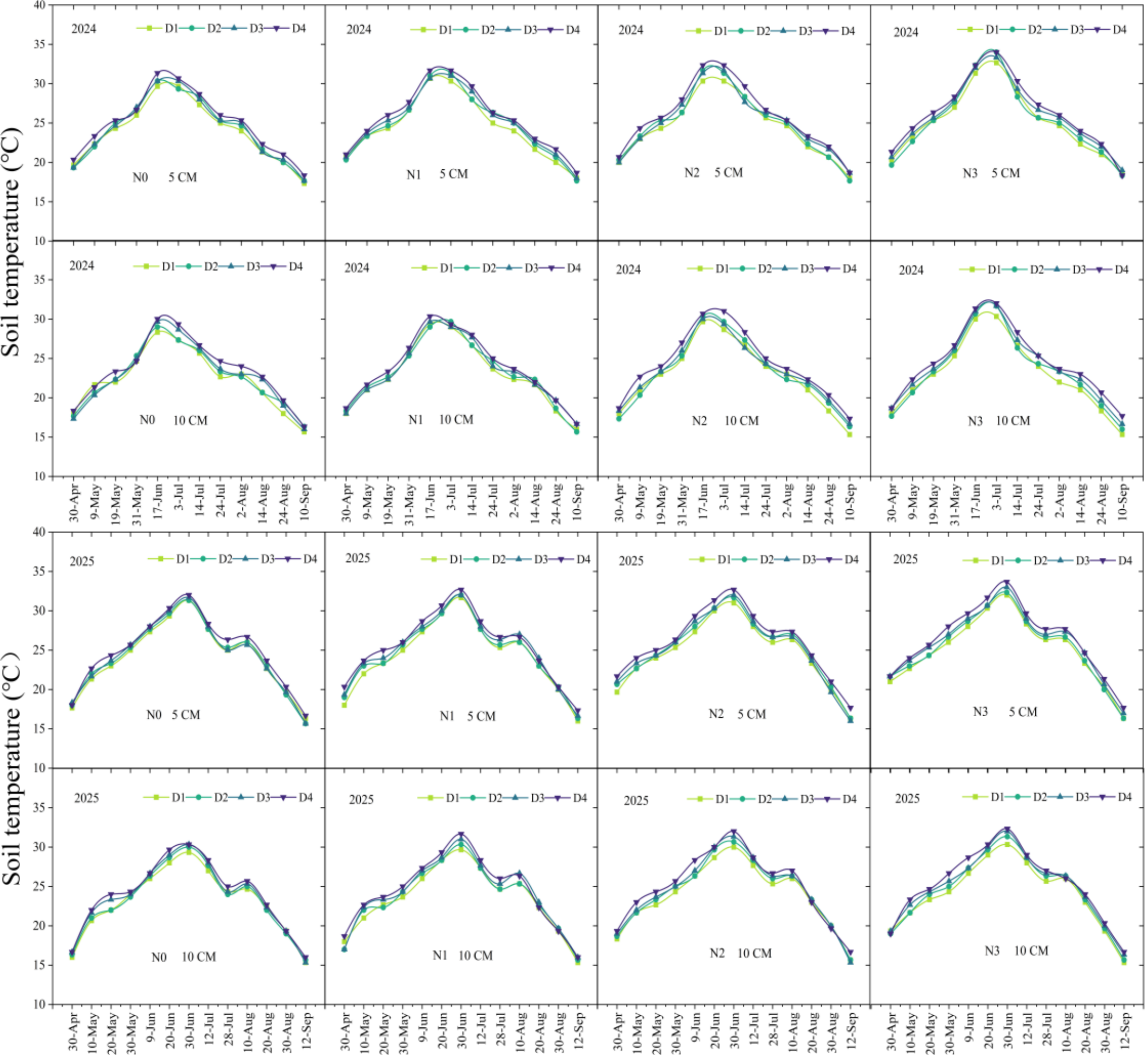
Seasonal dynamics of soil temperature at 5 cm and 10 cm soil depth under different planting densities and nitrogen application rates. D1, D2, D3, and D4 indicate the planting density of 90,000 plants ha^-1^,120,000 plants ha^-1^, 150,000 plants ha^-1^, and 180,000 plants ha^-1^. N0, N1, N2, and N3 represent nitrogen (N)application at 0 kg N ha^-1^, 180 kg N ha^-1^, 270 kg N ha^-1^, and 360 kg N ha^-1^.

During the maize growing season, soil temperature exhibited a seasonal pattern characterized by an initial increase followed by a gradual decline, peaked at the flowering stage (**Figure 1**). In both 2024 and 2025, the temperature at the 5 cm depth was consistently higher than that at the 10 cm depth. Overall, the average temperatures at the 5 cm and 10 cm layers were 24.98 °C and 23.28 °C in 2024, and 25.00 °C and 24.05 °C in 2025, respectively. The temperature trends were similar in both years, with each soil layer reaching its peak during the flowering stage. In 2024–2025, soil temperature under the N3 treatment was significantly higher than under N0 by 5.25% and 5.40% at the 5 cm layer, but showed no difference compared with N1 and N2. Similarly, the D4 treatment significantly increased temperature by 4.74% and 4.79% compared with D1 at the 5 cm layer, with no difference observed relative to D2 and D3. At the 10 cm layer in 2025, N3 increased temperature significantly by 5.23% compared with N0, but did not differ from N1 or N2, while D4 raised temperature by 5.53% and 4.51% compared with D1, with no difference from D2 and D3. However, no significant differences in soil temperature were observed among any treatments at any growth stage.

### 3.2 Soil water content

**Figure 2:**
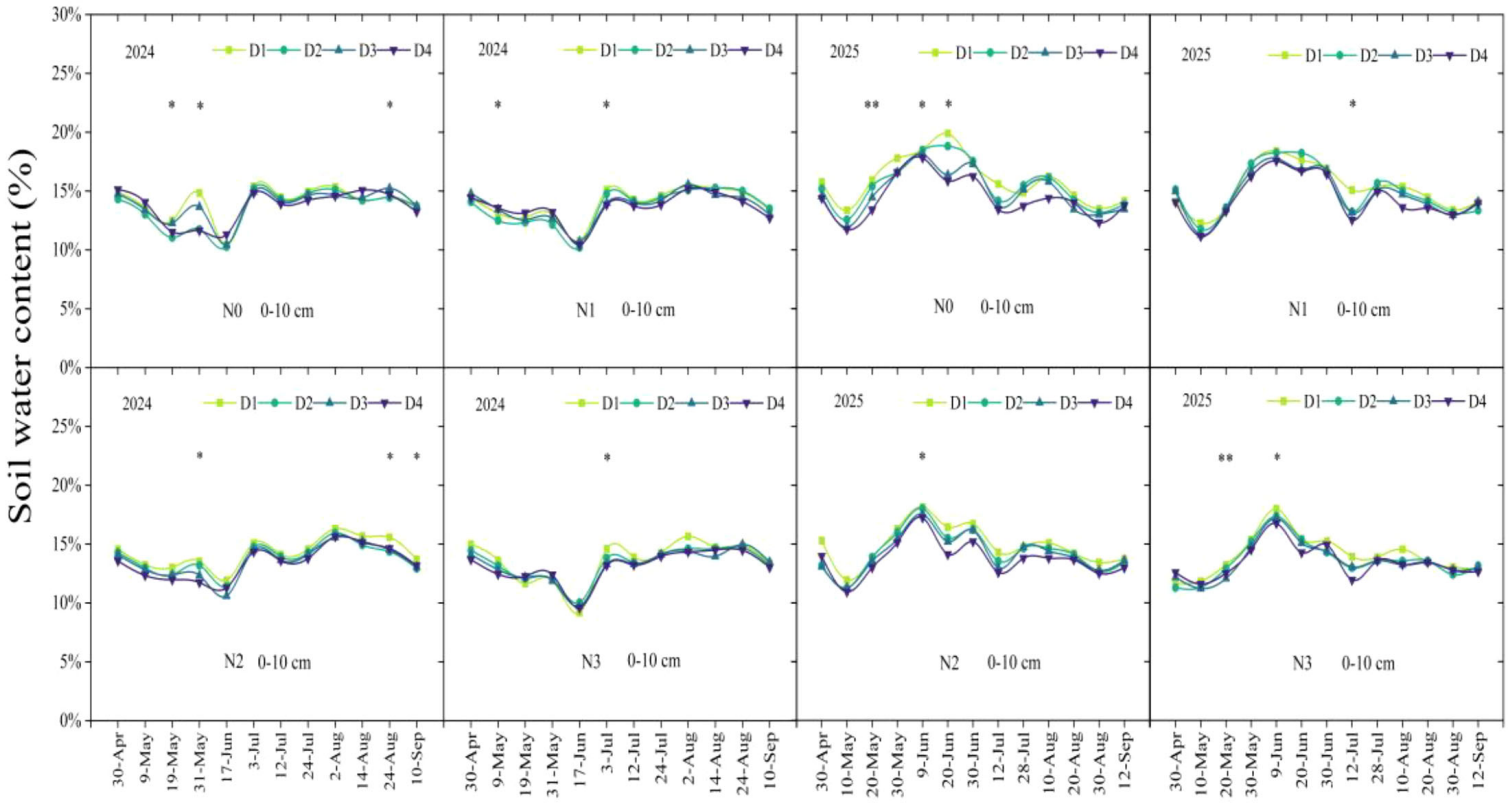
Dynamic changes of soil water content of 0-10 cm soil layer under different planting densities and nitrogen application rates. D1, D2, D3, and D4 indicate the planting density of 90,000 plants ha^-1^,120,000 plants ha^-1^, 150,000 plants ha^-1^, and 180,000 plants ha^-1^. N0, N1, N2, and N3 represent nitrogen (N)application at 0 kg N ha^-1^, 180 kg N ha^-1^, 270 kg N ha^-1^, and 360 kg N ha^-1^. *, *p* < 0.05; **, *p* < 0.01.

**Figure 3:**
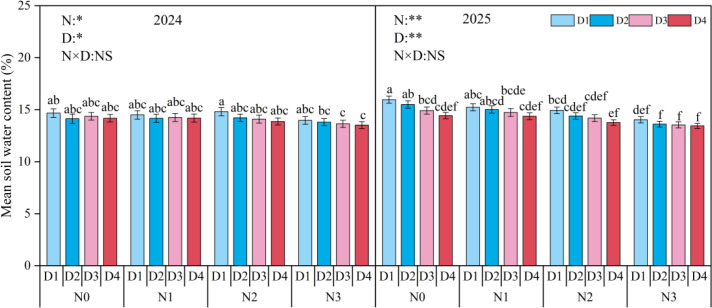
Mean soil water content (SWC) of 0-10 cm soil layer under different planting densities (D) and nitrogen(N) rates. D1, D2, D3, and D4 indicate the planting density of 90,000 plants ha^-1^,120,000 plants ha^-1^, 150,000 plants ha^-1^, and 180,000 plants ha^-1^. N0, N1, N2, and N3 represent nitrogen (N)application at 0 kg N ha^-1^, 180 kg N ha^-1^, 270 kg N ha^-1^, and 360 kg N ha^-1^. Different small letters indicate significant difference among treatments at *p* < 0.05. *, *p* < 0.05; **, *p* < 0.01; NS, no significant.

During the maize growing seasons in 2024-2025, soil water content in the 0–10 cm layer did not exhibit a consistent trend over time (**Figure 2**). Both nitrogen application rate and planting density had significant effects mean soil water content (**Figure 3**). In 2024, the average soil moisture under N0 was 3.62% higher than under N3, with no difference observed compared to N1 and N2. D1 was 2.73%, 2.89%, and 3.66% higher than D2, D3, and D4, respectively. In 2025, soil moisture under N0 was 6.14% and 11.31% higher than under N2 and N3, respectively, with no difference compared to N1. D1 was 4.84% and 7.41% higher than D3 and D4, respectively, and showed no difference compared to D2. Interaction analysis revealed that nitrogen application rate and planting density had a significant interactive effect on soil moisture only at the V8 stage, with no significant interaction observed during other growth stages.

### 3.3 Soil respiration rate

**Figure 4:**
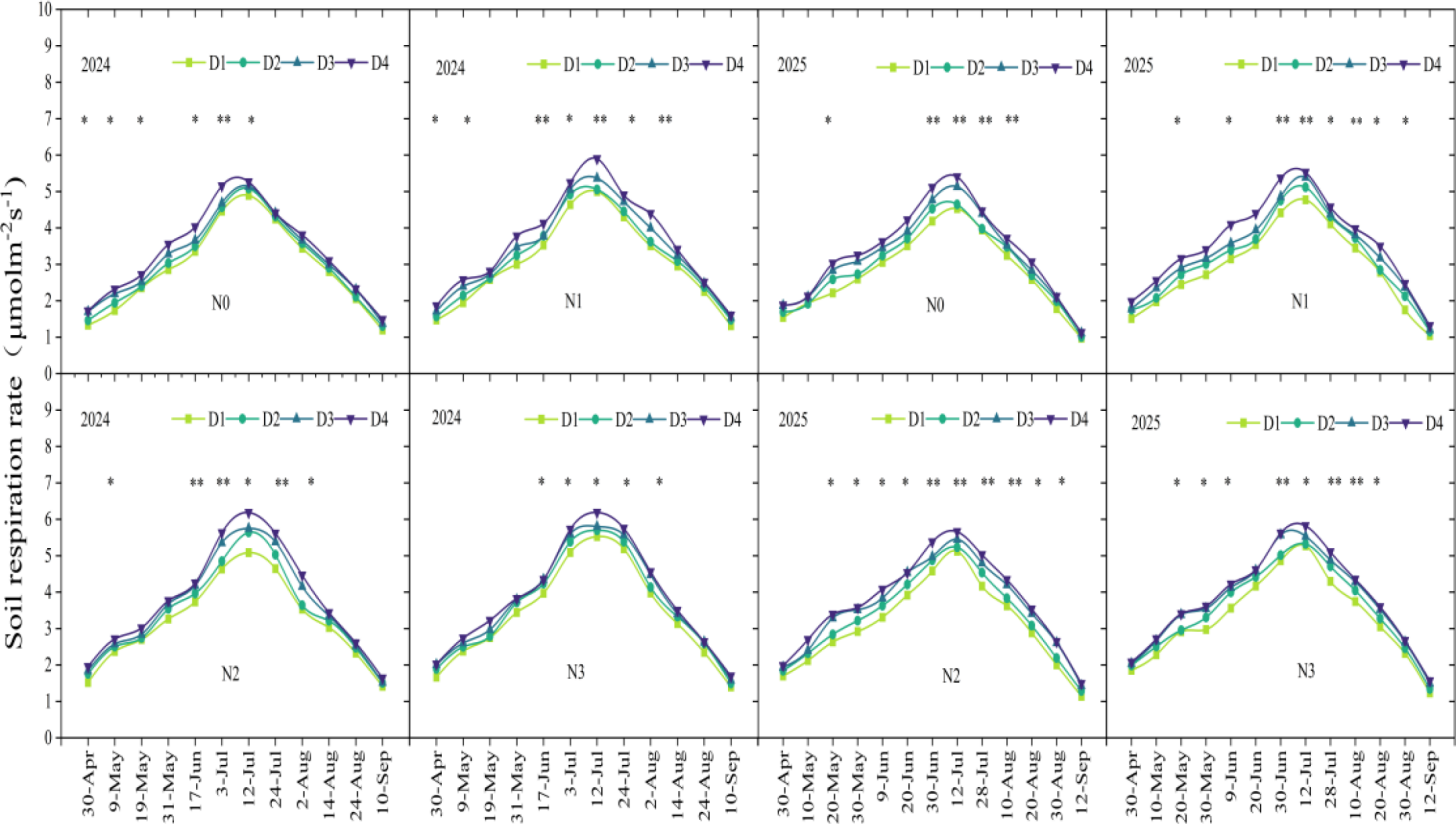
Temporal dynamics of soil respiration rate in response to planting densities and nitrogen rates. D1, D2, D3, and D4 indicate the planting density of 90,000 plants ha^-1^,120,000 plants ha^-1^, 150,000 plants ha^-1^, and 180,000 plants ha^-1^. N0, N1, N2, and N3 represent nitrogen (N)application at 0 kg N ha^-1^, 180 kg N ha^-1^, 270 kg N ha^-1^, and 360 kg N ha^-1^. *, *p* < 0.05; **, *p* < 0.01.

**Figure 5:**
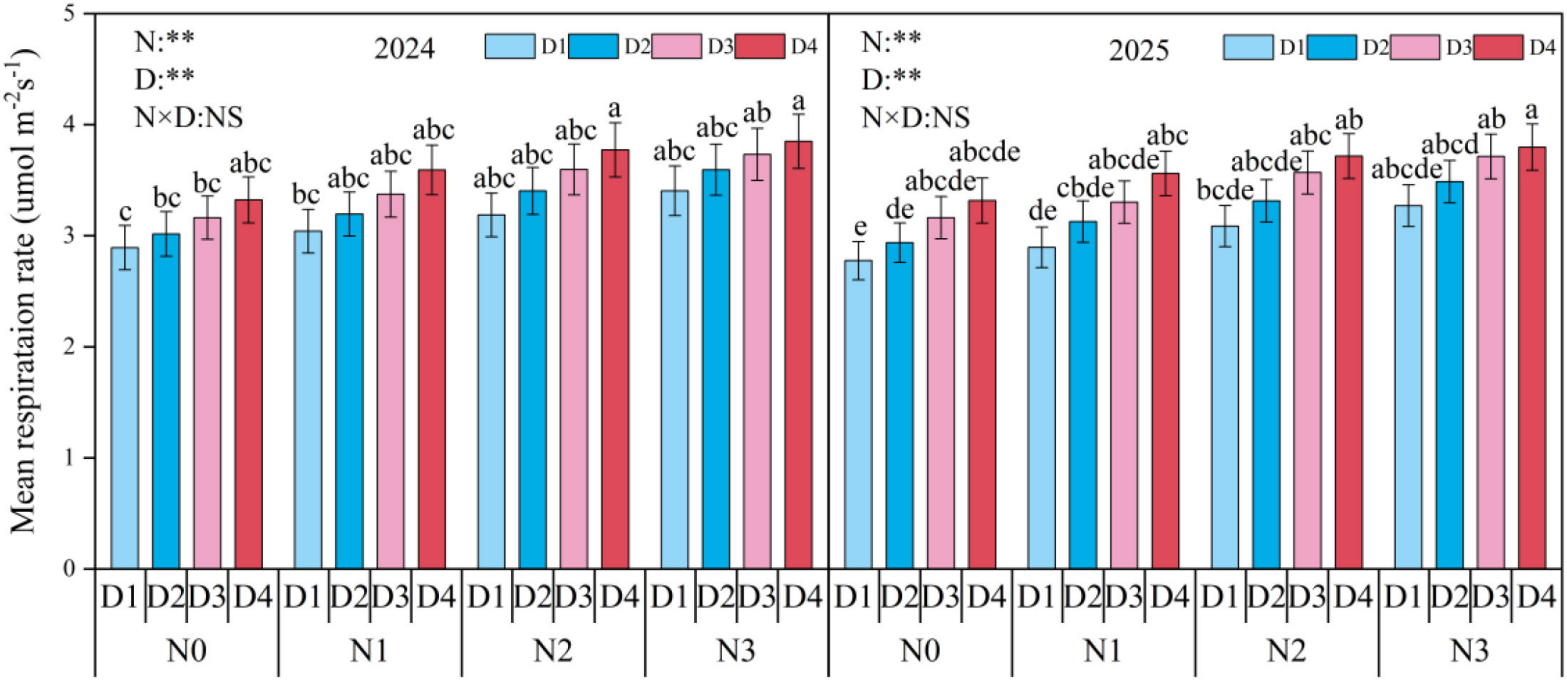
Mean soil respiration rate under different planting densities and nitrogen application rates. D1, D2, D3, and D4 indicate the planting density of 90,000 plants ha^-1^,120,000 plants ha^-1^, 150,000 plants ha^-1^, and 180,000 plants ha^-1^. N0, N1, N2, and N3 represent nitrogen (N)application at 0 kg N ha^-1^, 180 kg N ha^-1^, 270 kg N ha^-1^, and 360 kg N ha^-1^. Different small letters indicate significant difference among treatments at *p* < 0.05. *, *p* < 0.05; **, *p* < 0.01; NS, no significant.

During the maize growing season, soil respiration rates under different nitrogen application rates and planting densities are shown in the (**Figure 4**). Soil respiration exhibited distinct seasonal dynamics, with an overall trend similar to that of soil temperature. Soil respiration rates peaked in mid-July, reaching 6.19 μ mol m^-2^ s^-1^ in 2024 and 5.82 μ mol m^-2^ s^-1^ in 2025, respectively, and decreased to the minimum at maize harvest in September. The interaction of nitrogen application rate and planting density had significant effects on the soil CO_2_ emission rate at the peak growth period. Analysis with nitrogen application rate and planting density as main effects showed that both factors had significant effects on mean soil respiration rate (**Figure 5**). Across the two years, soil respiration under N0, N1, and N2 treatments decreased by 14.8% (*p* < 0.05), 9.6% (*p* < 0.05), and 4.2% (*p* > 0.05) compared to the N3 treatment, under the D1, D2, and D3 treatments decreased by 15.3% (*p* < 0.05), 10.1% (*p* < 0.05), and 5.0% (*p* > 0.05) compared to the D4 treatment.

### 3.4 Total carbon emission, grain yield, and carbon emission efficiency

**Figure 6:**
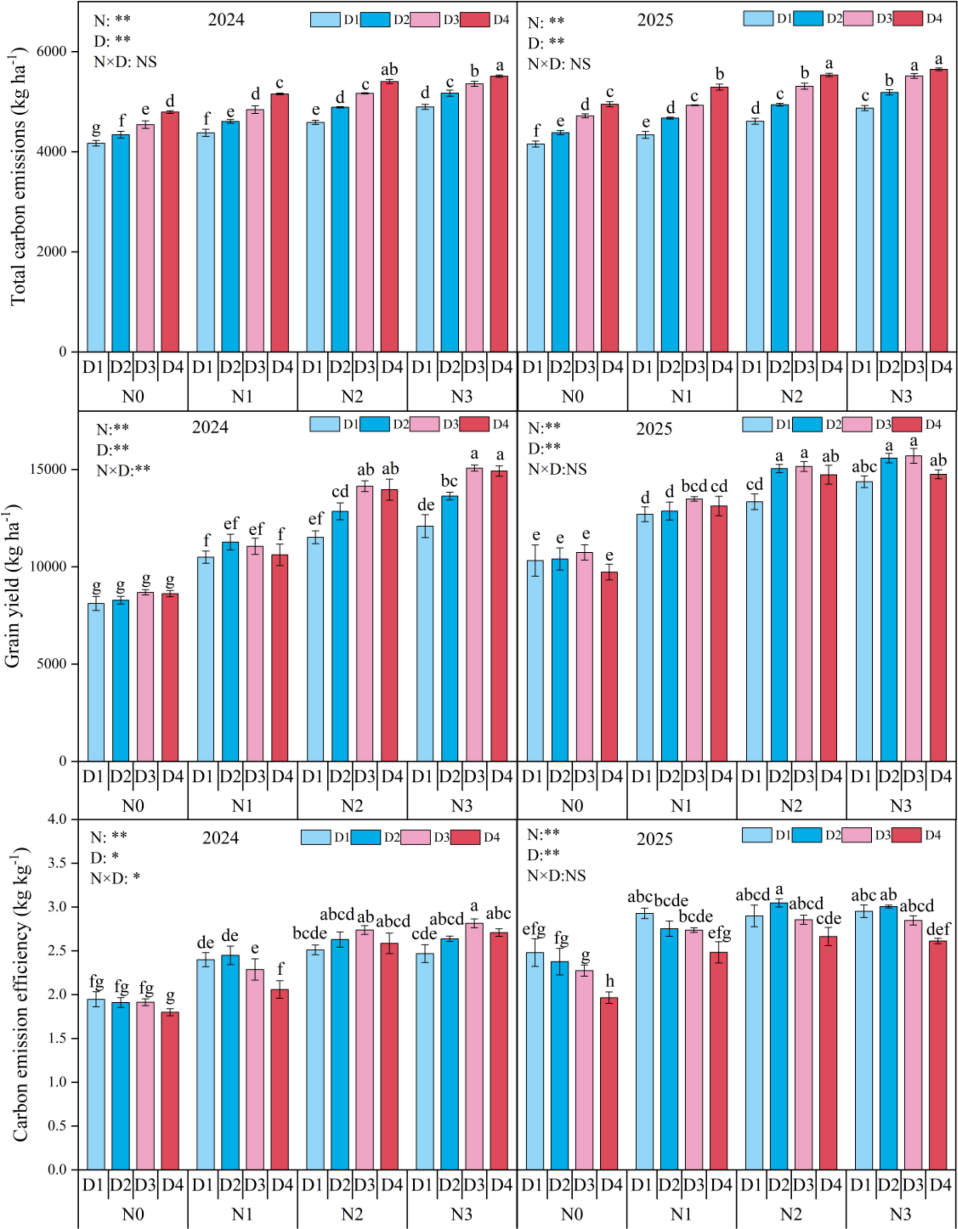
Total carbon emission, grain yield, and carbon emission efficiency under different planting densities (D) and nitrogen (N) rates. D1, D2, D3, and D4 indicate the planting density of 90,000 plants ha^-1^,120,000 plants ha^-1^, 150,000 plants ha^-1^, and 180,000 plants ha^-1^. N0, N1, N2, and N3 represent nitrogen (N)application at 0 kg N ha^-1^, 180 kg N ha^-1^, 270 kg N ha^-1^, and 360 kg N ha^-1^. Different small letters indicate significant difference among treatments at *p* < 0.05. *, *p* < 0.05; **, *p* < 0.01; NS, no significant.

During the maize growing seasons, nitrogen application rate and planting density significantly affected total soil CO_2_ emissions, grain yield, and carbon emission efficiency, while their interactive effect was not significant (**Figure 6**). Across the two years, total carbon emission under N0, N1, and N2 treatments decreased by 14.5% (*p* < 0.05), 9.4% (*p* < 0.05), and 4.1% (*p* < 0.05) compared to the N3 treatment, under the D1, D2, and D3 treatments decreased by 14.9% (*p* < 0.05), 9.7% (*p* < 0.05), and 4.5% (*p* > 0.05) compared to the D4 treatment. Grain yield under the N2 increased by 47.9% and 15.8%, under the N3 increased by 55.1% and 21.5% compared to the N0 and N1 treatments, respectively, under the D2 and D3 increased by 7.5% and 11.9% compared to the D1, respectively, but no significant with D4. Carbon emission efficiency under the N2 increased by 32.6% and 8.9%, under N3 increased by 33.9% and 10.0%, compared to the N0 and N1 treatments, no significant between N2 and N3; under the D1, D2, and D3 increased by 9.1%, 10.2% and 8.4%, compared to the D4, respectively.

### 3.5 Carbon balance

Across two years, the net ecosystem productivity (NEP) values for all treatments were positive, indicating that this farmland ecosystem acted as a net sink for atmospheric CO_2_ (**Table 1**). The NEP was increased with increasing rate of nitrogen application and planting densities. N2 increased NEP by 46.8% and 16.4% in 2024, by 47.5% and 10.8% in 2025, compared to N0 and N1, respectively, no significant with N3 in 2025. NEP under D2 increased by 8.6% compared to D4 in 2024, no significant difference was found among D2, D3, and D4 in 2025. Soil carbon sequestration potential of the ecosystem (Cs) under N1, N2, and N3 increased by 11.8%, 18.9%, and 28.7% in 2024, by 16.6%, 19.1%, and 20.3% in 2025, compared to the N0, respectively. Cs under the D1, D2, and D3 increased by 6.8%, 14.5%, and 16.5% in 2024, compared to D4.

**Table 1:** Carbon balance under different planting densities and nitrogen application rates.

Year	Treatment	DW(kg ha^-1^)	NPP(kg ha^-1^)	Rm(kg ha^-1^)	NEP(kg ha^-1^)	Cs
2024	N0D1	23100.70 ± 822.88ef	10395.32 ± 370.30ef	3605.99 ± 48.72g	6789.33 ± 403.51efg	2.50 ± 0.11defg
	N0D2	22593.87 ± 366.10f	10167.24 ± 164.74f	3753.09 ± 58.40f	6414.15 ± 155.08fg	2.34 ± 0.04fg
	N0D3	22662.17 ± 1153.48f	10197.98 ± 519.07f	3927.93 ± 66.16e	6270.04 ± 482.97g	2.24 ± 0.10g
	N0D4	23698.13 ± 1445.76def	10664.16 ± 650.59def	4145.02 ± 23.18d	6519.14 ± 661.07fg	2.23 ± 0.14g
	N1D1	27360.60 ± 892.232cd	12312.27 ± 401.50cd	3785.87 ± 62.42f	8526.40 ± 413.43cd	2.82 ± 0.11bcd
	N1D2	27414.53 ± 1479.22cd	12336.54 ± 665.65cd	3983.55 ± 32.01e	8352.99 ± 695.79de	2.68 ± 0.16cdef
	N1D3	26777.50 ± 1040.44cde	12049.88 ± 468.20cde	4187.01 ± 66.30d	7862.87 ± 406.40defg	2.49 ± 0.06defg
	N1D4	27776.73 ± 1346.93c	12499.53 ± 606.12c	4458.70 ± 15.59c	8040.83 ± 592.73def	2.42 ± 00.11efg
	N2D1	27980.30 ± 1163.54c	12591.14 ± 523.60c	3966.01 ± 35.37e	8625.12 ± 489.20bcd	2.74 ± 0.09cde
	N2D2	29410.27 ± 1469.48bc	13234.62 ± 661.27bc	4227.99 ± 11.18d	9006.63 ± 655.70bcd	2.71 ± 0.13cde
	N2D3	32988.17 ± 624.63b	14844.68 ± 281.08b	4469.19 ± 10.04c	10375.49 ± 272.48b	2.87 ± 0.05abc
	N2D4	32914.60 ± 1821.42b	14811.57 ± 819.64b	4673.40 ± 37.85ab	10138.17 ± 822.13bc	2.74 ± 0.15cde
	N3D1	30488.17 ± 1117.58bc	13719.68 ± 502.91bc	4234.13 ± 44.34d	9485.55 ± 475.10bcd	2.80 ± 0.09bcd
	N3D2	32741.73 ± 1461.31b	14733.78 ± 657.59b	4471.37 ± 52.69c	10262.41 ± 630.92bc	2.85 ± 0.11bcd
	N3D3	38013.67 ± 1038.74a	17106.15 ± 467.43a	4633.54 ± 43.33b	12472.61 ± 442.72a	3.19 ± 0.07a
	N3D4	38477.73 ± 1508.24a	17314.98 ± 678.71a	4765.41 ± 19.80a	12549.57 ± 661.81a	3.14 ± 0.11ab
2025	N0D1	25395.83 ± 563.82fg	11428.13 ± 253.72fg	3592.36 ± 52.00f	7835.76 ± 225.38d	2.75 ± 0.05d
	N0D2	26428.93 ± 919.60f	11893.02 ± 413.82f	3789.49 ± 35.73e	8103.53 ± 446.90d	2.72 ± 0.12d
	N0D3	25657.00 ± 599.75fg	11545.65 ± 269.87fg	4080.64 ± 34.50d	7465.01 ± 238.24d	2.45 ± 0.04e
	N0D4	23352.47 ± 486.42g	10508.61 ± 218.89g	4279.67 ± 43.88c	6228.94 ± 183.34e	2.12 ± 0.03f
	N1D1	30399.83 ± 1280.56e	13679.93 ± 576.25e	3751.20 ± 59.72e	9928.73 ± 537.02bc	3.15 ± 0.10ab
	N1D2	30660.40 ± 1032.25e	13797.18 ± 464.51e	4041.13 ± 16.72d	9756.05 ± 448.71c	2.95 ± 0.09bcd
	N1D3	31643.50 ± 569.61de	14239.58 ± 256.33de	4263.01 ± 6.28c	9976.56 ± 261.84bc	2.89 ± 0.06cd
	N1D4	31891.40 ± 708.27de	14351.13 ± 318.72de	4576.27 ± 51.57b	9774.86 ± 370.82c	2.71 ± 0.09d
	N2D1	32496.70 ± 571.03cde	14623.52 ± 256.96cde	3986.23 ± 51.85d	10637.29 ± 300.20abc	3.18 ± 0.09ab
	N2D2	35380.40 ± 503.40ab	15921.18 ± 226.53ab	4271.42 ± 24.52c	11649.76 ± 250.73a	3.22 ± 0.06a
	N2D3	33959.83 ± 1062.65abcd	15281.93 ± 478.19abcd	4591.96 ± 52.31b	10689.96 ± 492.73abc	2.88 ± 0.10cd
	N2D4	34424.80 ± 838.46ab	15491.16 ± 377.31ab	4784.86 ± 29.75a	10706.30 ± 402.72abc	2.80 ± 0.08d
	N3D1	33586.30 ± 953.22bcd	15113.84 ± 428.94bcd	4213.34 ± 43.92c	10900.50 ± 472.47abc	3.11 ± 0.12abc
	N3D2	35490.13 ± 300.76ab	15970.56 ± 135.34ab	4485.03 ± 46.31b	11485.53 ± 115.09a	3.08 ± 0.03abc
	N3D3	36178.00 ± 707.26a	16280.10 ± 318.27a	4768.72 ± 41.48a	11511.38 ± 289.79a	2.95 ± 0.04bcd
	N3D4	35409.40 ± 789.08ab	15934.23 ± 355.09ab	4884.07 ± 23.79a	11050.16 ± 338.55ab	2.82 ± 0.05d

DW, aboveground biomass; NPP, net primary productivity; Rm, carbon emission by soil microbial heterotrophic respiration; NEP, net ecosystem productivity; Cs, Soil carbon sequestration potential. N0, N1, N2, and N3 represent nitrogen (N) application at 0 kg N ha^-1^, 180 kg N ha^-1^, 270 kg N ha^-1^, and 360 kg N ha^-1^. D1, D2, D3, and D4 indicate the planting density of 90,000 plants ha^-1^, 120,000 plants ha^-1^, 150,000 plants ha^-1^, and 180,000 plants ha^-1^. Different small letters indicate significant difference among treatments at *p<* 0.05.

### 3.6 Regression analysis of soil respiration rate in relation to soil moisture and temperature

**Figure 7:**
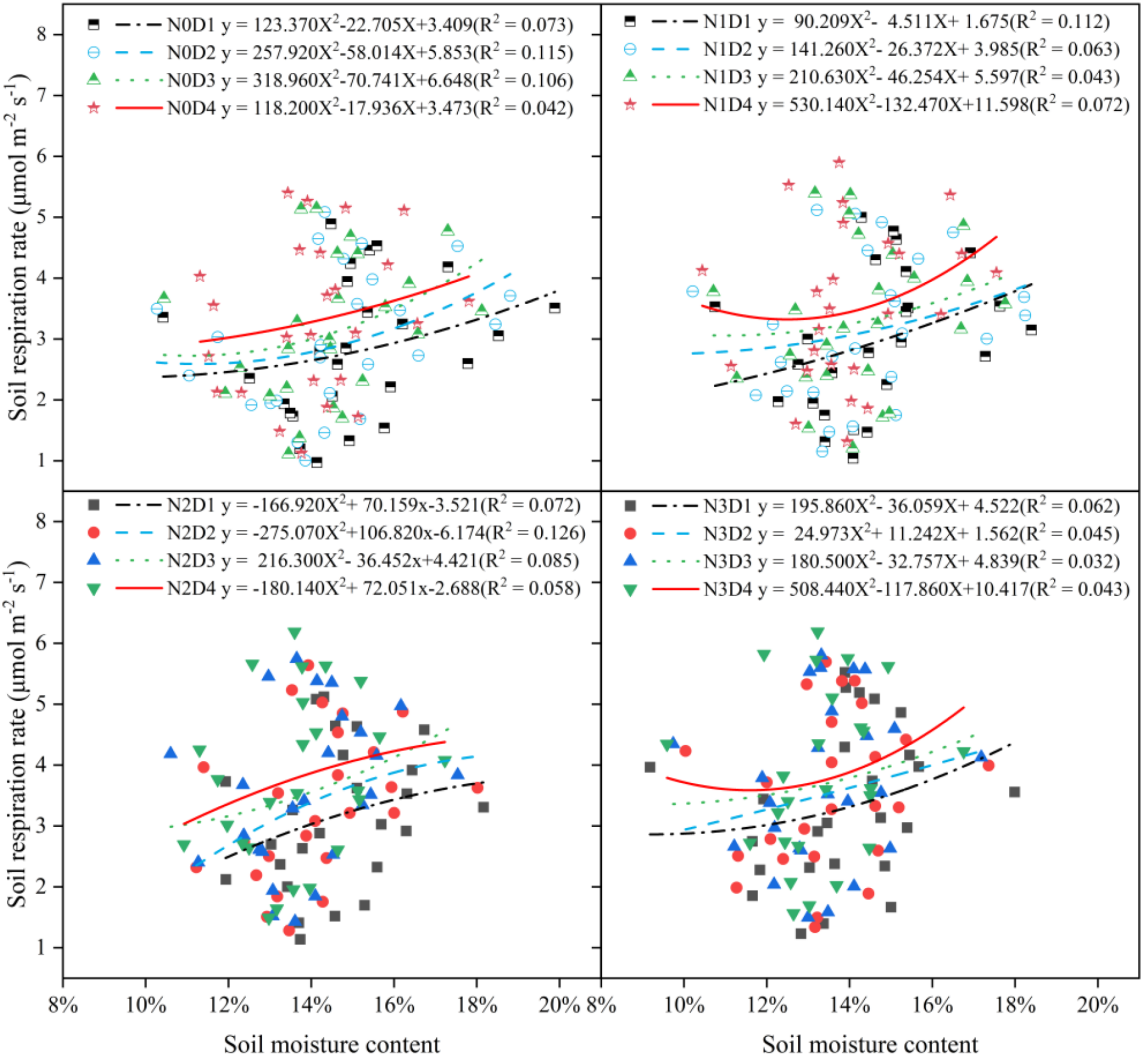
Fitting analysis of the relationship between soil respiration rate and soil water content under different planting densities and nitrogen application rates.

**Figure 8:**
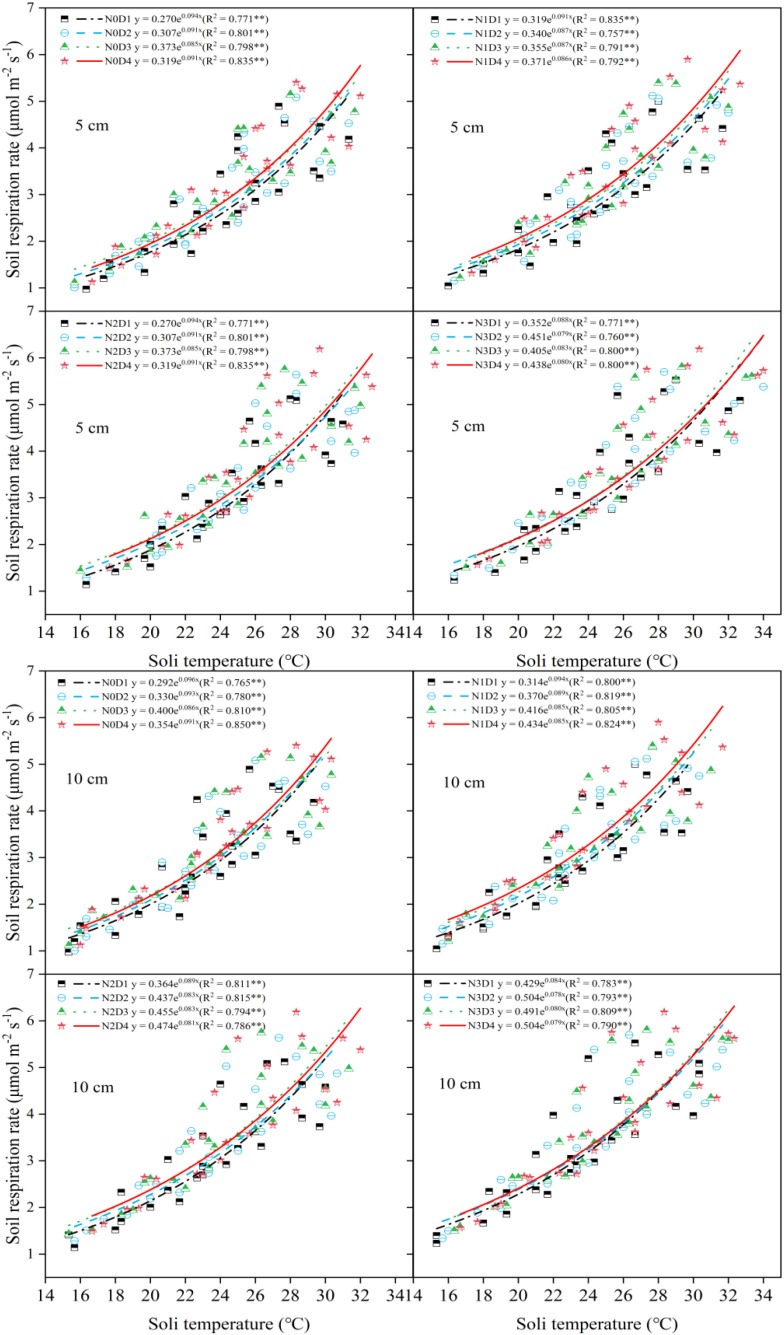
Fitting analysis of the relationship between soil respiration rate and soil temperature under different planting densities and nitrogen application rates.

The relationship between soil respiration rate and soil water content was analyzed using nonlinear fitting methods (**Figure 7**). The fitting equations between soil respiration rate and soil moisture under different treatments did not reach a significant level. Soil water content could explain 3.2% to 12.6% of the variation in soil respiration emission rate during the whole period. The relationship between soil respiration rate and soil temperature was analyzed using exponential fitting within nonlinear regression (**Figure 8**). Soil respiration rate was significantly correlated with soil temperature under different treatments at different soil depth. Under varying nitrogen application rates and planting densities, soil temperature could explain 75.7% to 84.9% of the variation in soil respiration rate, which was higher than the explanatory power of soil moisture.

## 4 Discussion

### 4.1 Soil carbon emissions under different planting density and nitrogen rates

Soil temperature and moisture are key factors influencing soil respiration (Chen et al., 2024). In this study, soil respiration rate exhibited a distinct single-peak curve, and its seasonal variation showed a highly significant positive correlation with soil temperature. Moreover, previous studies have indicated an exponential relationship between soil temperature and soil carbon emission rate, which is consistent with the findings of the present study (Wang et al., 2017). The underlying reason may be that soil temperature is the key factor regulating soil respiration in this region; when the respiration rate peaks at the highest temperature, soil microbial activity, enzyme activity, and root activity are also at their maximum (Zhang et al., 2020). This period coincides with the vigorous growth stage of maize, thereby increasing the respiratory flux (Meyer et al., 2018). As temperature decreases and plant growth senesces, the activity of these biological components declines, leading to a gradual reduction in the soil carbon emission rate (Prudence et al., 2021). In contrast, no relationship was observed between soil respiration rate and soil moisture. This may be because when soil water content ranges between the wilting coefficient and field capacity, its impact on microorganisms and crop growth is minimal, and consequently does not affect soil respiration (Manzoni et al., 2012).

Previous studies have established that planting density and nitrogen fertilizer are critical factors limiting maize growth (Tian et al., 2022). Increasing planting density and nitrogen application are important measures to enhance crop yield; however, they also significantly affect soil carbon emissions (Cao et al., 2025). This study found that soil carbon emissions increased with increasing planting density and nitrogen application rate (Jiang et al., 2025). Nitrogen addition promotes crop growth, increases leaf area index and photosynthetic capacity, and enhances the redistribution of belowground carbohydrates, thereby boosting root vigor and activity, ultimately increasing soil carbon emissions (Yuan et al., 2023). Increasing planting density intensifies plant competition for soil water and nutrients, stimulates root metabolic activity, and leads to higher respiratory consumption by roots to support nutrient uptake (Sun et al., 2018). Concurrently, high-density populations increase soil biomass and soil respiration rates. The increase in vegetation and litter reduces surface evaporation and mitigates soil water loss, providing favorable moisture conditions for microbial activity, thereby synergistically promoting soil respiration (Escolar et al., 2015). This aligns with the conclusion from studies on high-yield fields that “optimized hydrothermal conditions promote respiration” (Li et al., 2024). The interactive effect of planting density and nitrogen application rate on soil respiration was significant only during the peak growth period. This may be because the synergistic effect of density and nitrogen is amplified during periods of high nutrient demand and intense resource competition (Pico et al., 2021), whereas during other growth stages, this effect is diluted or offset by compensatory mechanisms (Zhou et al., 2025). This aligns with relevant research on the Loess Plateau, which found that optimizing nitrogen management under plastic film mulching can significantly improve the soil quality index and enzyme activities, thereby regulating crop growth conditions and resource use efficiency (Zhou et al., 2025). Nitrogen and density may act through partially overlapping pathways, such that the effect of one factor can compensate for or mask the effect of the other (Zhang et al., 2025). Furthermore, nitrogen application alleviates nitrogen stress under high planting density, promotes canopy growth and root development, and consequently diminishes the differences in carbon emissions among treatments, rendering the interaction non-significant during other growth periods (Ciampitti and Vyn, 2011).

### 4.2 Grain yield and carbon balance under different planting density and nitrogen rates

Planting density and nitrogen application rate are two key factors regulating maize growth and yield (Xu et al., 2017). This study found that a moderate increase in planting density significantly enhanced maize yield; however, when the density became excessively high, the yield-enhancing effect tended to diminish. This is likely because excessively high density intensifies competition among individual plants, potentially leading to uneven distribution of light, water, and nutrients, which limits further yield improvement (Zhang et al., 2022). Similarly, the application of nitrogen fertilizer significantly promoted maize yield, but excessive nitrogen application did not bring a corresponding yield increase. This is primarily because excessive nitrogen input disrupts the synergistic balance of nutrients within the plant, leading to metabolic disorders, which in turn weakens photosynthetic efficiency and ultimately restricts further yield gains (Ye et al., 2025).

This study assessed the carbon economic benefits of a maize cropping system under varying planting densities and nitrogen (N) application rates, using carbon emission efficiency (CEE), net ecosystem productivity (NEP), and carbon sequestration potential (Cs) as key indicators. CEE reflects the trade-off between soil carbon emissions and grain yield. Although higher planting densities and N rates increased total carbon emissions, they also enhanced yield, thereby improving CEE (Xu et al., 2017; Cao et al., 2025). In this study, CEE was significantly higher under N2 and N3 N treatments compared to N0 and N1, and higher under D3 planting density than under D4, primarily due to greater grain yield per unit of CO_2_ emitted (Yang et al., 2021). NEP, which represents the net carbon budget, showed a synergistic relationship with Cs. All treatments exhibited positive NEP values, confirming that the irrigated oasis farmland functions as a carbon sink (Sun et al., 2025). Notably, increases in NEP and Cs diminished as N application rose beyond the N2 level, where gains plateaued or declined. This suggests that moderate N application boosts net primary productivity (NPP) and Cs despite increasing emissions (Ma et al., 2020). However, excessive N saturates photosynthesis while stimulating root and microbial respiration, limiting further gains in Cs (Hu and Dusenge, 2025). Regarding planting density, NEP and Cs under D2 and D3 were comparable to or higher than those under D4, indicating that moderate densities achieve a favorable balance between low emissions and high productivity (Xu et al., 2017). The synchronized variation between NEP and Cs suggests that both excessive density and N application reduce yield and ecological carbon sequestration (Bai and Ding, 2024). These results imply that crop yield and carbon sequestration responses to density and N are governed by an ecological threshold, transitioning from facilitation to competition (Cao et al., 2025). Within the optimal range (D2-D3 density, N2 rate), increasing inputs improves the carbon source-sink balance, enhances resource capture, and boosts both yield and sequestration (Zhang et al., 2025). Beyond this threshold (D4 density, N3 rate), canopy saturation and futile respiration disrupt the carbon budget, reducing both NEP and CEE (Skinner, 2013). The optimal management identified 12,000-15,000 plants ha^-1^ and 270 kg N ha^-1^-effectively balances emissions and sequestration, delivering both ecological and economic benefits.

While this study has provided preliminary insights into the effects of nitrogen application rate and planting density on carbon emissions, certain limitations remain, and several mechanistic questions have yet to be fully addressed. Future research will focus on elucidating the underlying mechanisms from the perspectives of soil respiration components and microbial communities.

## 5 Conclusion

Soil respiration exhibited a seasonal pattern characterized by an initial increase followed by a gradual decline, peaked at the flowering stage, and significant corrected with soil temperature. Soil CO_2_ emission under N0, N1, and N2 treatments were lower than under the N3 treatment, under the D1, D2, and D3 treatments were lower than under the D4 treatment. Grain yield and carbon emission efficiency under the N2 and N3 were higher than under the N0 and N1 treatments, under the D3 and D2 were higher than under the D1. NEP under the N2 treatment was higher than under N0 and N1, but no significant with difference with N3. NEP under D2 was higher than under D4 in 2024, no significant difference was found among D2, D3, and D4 in 2025. Overall, the combination of a nitrogen application rate about 270 kg ha^-1^ and a planting density of 120,000–150,000 plants ha^-1^ can simultaneously enhance yield, reduce carbon emissions, and strengthen carbon sequestration potential. This approach achieves low-carbon and high-yield maize production, serving as a recommended management strategy for intensive maize field in the south Xinjiang.

## Data Availability

The raw data supporting the conclusions of this article will be made available by the authors, without undue reservation.
